# Secondary Metabolites in *Ramalina terebrata* Detected by UHPLC/ESI/MS/MS and Identification of Parietin as Tau Protein Inhibitor

**DOI:** 10.3390/ijms17081303

**Published:** 2016-08-18

**Authors:** Alberto Cornejo, Francisco Salgado, Julio Caballero, Reinaldo Vargas, Mario Simirgiotis, Carlos Areche

**Affiliations:** 1 Facultad de Medicina, Escuela de Tecnología Médica, Universidad Andrés Bello, Sazié 2315, Primer Piso, Santiago 8370092, Chile; 2 Departamento de Química, Facultad de Ciencias, Universidad de Chile, Ñuñoa, Santiago 8320000, Chile; fsalgado@ug.uchile.cl; 3 Centro de Bioinformática y Simulación Molecular, Facultad de Ingeniería, Universidad de Talca, 2 Norte 685, Casilla 721, Talca 3460000, Chile; jcaballero@utalca.cl; 4 Departamento de Biología, Universidad Metropolitana de Ciencias de la Educación, Avda. Jose Pedro Alessandri 774, Ñuñoa, Santiago 8320000, Chile; reinaldo.vargas@umce.cl; 5 Laboratorio de Productos Naturales, Instituto de Farmacia, Facultad de Ciencias, Universidad Austral de Chile, Casilla 567, Valdivia 5090000, Chile; mario.simirgiotis@gmail.com

**Keywords:** Alzheimer’s disease, docking, Ramalina, tau protein, lichens, parietin, UHPLC/MS

## Abstract

Liquid chromatography coupled with mass spectrometry is an outstanding methodology for fast analysis of phenolic compounds in biological samples. Twenty two compounds were quickly and accurately identified in the methanolic extract of the Antarctic lichen *Ramalina terebrata* for the first time using ultra high pressure liquid chromatography coupled with photodiode array detector and high resolution mass spectrometry (UHPLC-PDA-Q/Orbitrap/MS/MS). In addition, the extract and the four compounds isolated from this species were tested for the inhibitory activity of tau protein aggregation, which is a protein involved in Alzheimer’s disease (AD). All compounds showed null activity with the exception of parietin, which it was able to inhibit aggregation process of tau in a concentration range between 3 µg/mL (10 µM) to 28 µg/mL (100 µM). In addition, we show how parietin interact with tau ^306^VQIVYK^311^ hexapeptide inside of the microtubule binding domain (4R) with the help of molecular docking experiments. Finally, the constituents present in the methanolic extract could possibly contribute to the established anti-aggregation activity for this extract and this in-depth analysis of the chemical composition of *R. terebrata* could guide further research into its medicinal properties and potential uses.

## 1. Introduction

Lichens are symbiotic associations between heterotrophic fungi and algae and/or cyanobacteria. A peculiarity of lichen is its remarkable ability to tolerate extreme atmospheric conditions such as low temperatures in polar zones including the Arctic and Antarctic regions; these regions are very cold; the coldest temperature ever known on earth (−129 °F) was recorded in Antarctica. These environmental conditions are responsible for the diversity of secondary metabolites produced in lichens [1,2,3,4,5]. Lichen substances are mainly synthesized via poly-malonyl, shikimate, and mevalonic acid pathway, which have afforded several interesting and unique phenolic structures such as dibenzofurans, depsides, depsidones, depsones, quinones and pulvinic acid derivatives [1,2,3,4,5,6,7].

Alzheimer´s disease is the most common form of dementia. There are two main proteins involved, β-amyloid protein and microtubule-associated tau protein. Both proteins are characterized by the deposition of plaques and neurofibrillary tangles, respectively [8]. Physiologically, tau protein is involved in axonal transport and microtubule stability. However, once tau is hyper-phosphorylated, it detached from microtubules and starts to form aggregates in soma and dendrites of neuron cells [9]. Tau is an unfolded protein whose structure has two fibril-forming motif, ^275^VQIINK^280^ and ^306^VQIVYK^311^ [10]. Besides, in order to form the fibrillar structure of tau, is required the addition of polyanions such as heparin which suggest an important role of electrostatic interaction to form both fibrils and aggregates [11]. These two motifs are within the microtubule binding domain of tau and are prone to forming a cross β structure [12,13].

The determination of amyloid-like structure reveals the presence of moieties involved in β sheets pair formation [14], this “steric zipper” is formed from short self-complementary segments of the amyloid [15]. Thus, this formation is a central part of proto-filaments, whereas the rest of the protein remains unfolded outside of the main axis [16]. The Q-exactive focus is a newly released hybrid high resolution mass spectrometer used for metabolomics analysis including pesticides, herbicides, drugs, antibiotics, small peptides and several other organic molecules [17,18,19,20,21]. The hyphenated Q-exactive focus instrument combines ultra high pressure liquid chromatography coupled with photodiode array detector (UHPLC-PDA) with an orbital trap and a high-resolution collision cell, which allows high resolution MS^n^ fragments [17,18,19,20,21].

On the other hand, molecular docking is a computational method used for the prediction of ligand-receptor interactions and is an important tool for rational drug design [22]. Today, molecular docking is the most important theoretical method to determine the orientation of the ligands inside a binding site. Particularly, the challenges of molecular docking are the following: the prediction of ligands proper orientation, the prediction of the binding energies and the prediction of novel, effective drugs by using the structural knowledge obtained from the models [22,23,24]. Several examples using these computational methods have been already reported [23,24,25,26,27,28].

The present work describes the UHPLC chromatographic fingerprints plus the isolation of the main secondary metabolites together with the tau aggregation inhibitory activity of methanolic extract of *Ramalina terebrata*. Hook. and Taylor. Based on the reported activity of fulvic acid [29] we decided to investigate parietin, in order to demonstrate its capacity to inhibit tau protein aggregation. Moreover, we modeled the structure of the complex between parietin and fibril-forming motif VQIVYK of tau using docking experiments. Hence, we are able to demonstrate that parietin is able to create hydrogen bonds (HB) with lysine residues.

## 2. Results and Discussion

**Figure 1:**
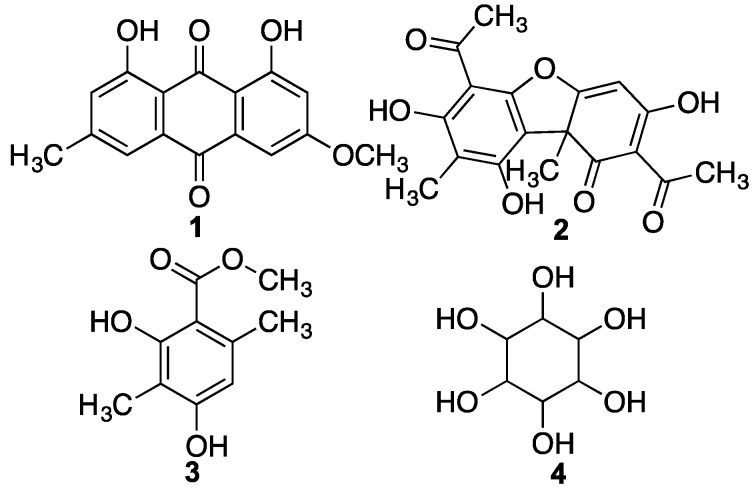
Structures of compounds **1**–**4** isolated from Antartic lichen *R. terebrata*.

From the methanolic extract, the following compounds were isolated: parietin **1**, usnic acid **2**, atraric acid **3** and inositol **4** (Figure 1) using a combination of chromatographic techniques [30].

**Figure 2:**
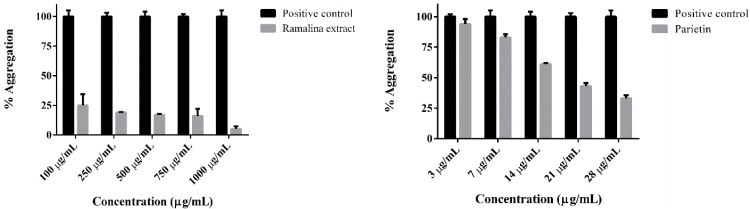
Tau aggregation process inhibited by both Ramalina extract and parietin. Black and grey bars represent positive control (aggregation) and inhibition respectively. A paired samples-*t*-test was conducted in order to compare control (aggregation) and tau inhibitors (Ramalina extract and parietin). There was a significant difference for both Ramalina extract *t* (4) = 25, *p* < 0.05 and parietin *t* (4) = 3.223, *p* < 0.05 (data are represented as Mean ± SEM).

Initially, the methanolic extract was screened by Thioflavin T (ThT) at concentration ranging from 100 to 1000 µg/mL and the results reveled that at 1000 µg/mL the inhibitory activity against aggregation process of tau protein was almost complete (Figure 2). Therefore, we have performed the isolation of lichen substances from this extract for further testing of this activity. All isolated compounds were tested in ThT fluorescence assay, since it has been demonstrated that ThT is able to bind to fibrils from both synthetic and biological sources [31,32]. None of these compounds were active to prevent tau aggregation with the exception of parietin (Figure 2).

Parietin, an orange anthraquinone pigment, is a metabolite very common in the family Teloschistaceae. Several biological activities for this compound have been summarized [1,4]. Besides, it is noteworthy to mention that parietin isolated from *Xanthoria parietina* (Linnaeus) Theodor Fries showed antibacterial activity against *S. aureus* (ATCC and clinical isolate strains), antifungal activity towards *Rhizoctonia solani*, *Botrytis cinerea* and *Candida albicans*. In contrast, parietin did not show any effect regarding anticancer and antiproliferative activity [2,3,4]. Gauslaa and Ustvedt reported that parietin may reduce the effect of UV radiations [2,3,4]. However, there is no information regarding its capacity to inhibit tau aggregation process. Furthermore, according to our knowledge, there is no published data on anti-aggregation properties of lichen compounds.

The aggregation assay was performed using fragment 4R of the protein tau as positive control. Once we tested parietin by ThT, our results showed that parietin was able to inhibit aggregation process of tau in a concentration range between 3 µg/mL (10 µM) to 28 µg/mL (100 µM) (Figure 2) showing a dose-response effect. The inhibitory effect of parietin at 28 µg/mL was by 75%.

Some compounds have been described for their anti-aggregating effect against over either amyloid-β or tau protein [33,34,35]. In addition, anthraquinones compounds such as daunorubicin, adriamycin and emodin inhibit tau aggregation and also diminish paired helical filaments in cells [36]. A previous report has shown that emodin, an anthraquinone related to parietin has profound effects on aggregation process of tau protein [36], this difference could be because parietin has a methoxy group at C-3 position instead of hydroxyl group of emodin.

**Figure 3:**
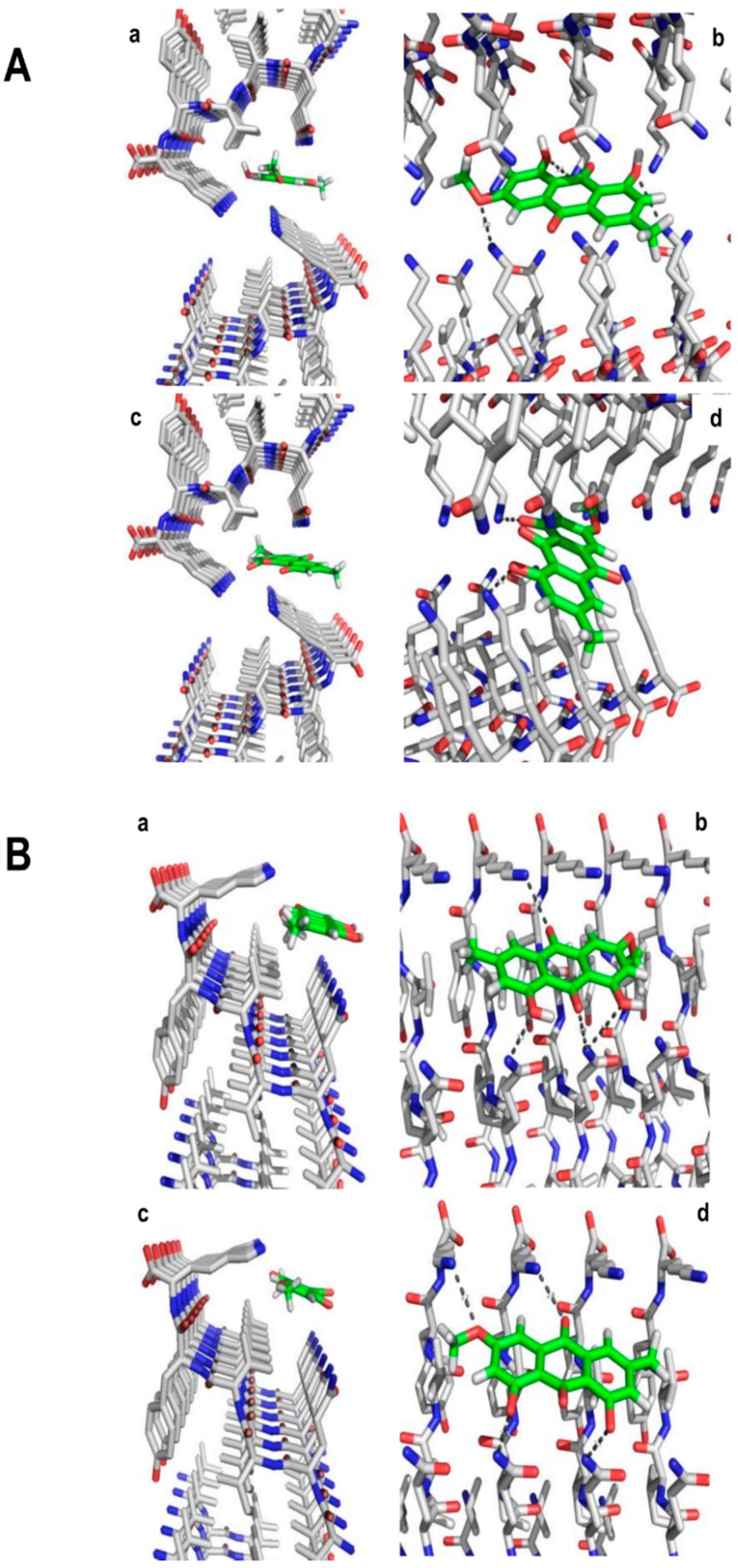
(**A**) Docking of parietin in protonation state P1 in the interface of two poly-^306^VQIVYK^311^ hexapeptide zippers (**a**,**c**-left). Hydrogen bounds (HBs) between parietin and lysine residues are indicted as broken lines in the perpendicular view (**b**,**d**-right); (**B**) Docking of parietin in protonation state P2 in the interface of two poly-^306^VQIVYK^311^ hexapeptide zippers (**a**,**c**-left). Hydrogen bounds (HBs) between parietin and lysine residues are indicated as broken lines in the perpendicular view (**b**,**d**-right).

There are previous evidences that negatively charged molecules such as orange-G bind specifically to the lysine residues of tau fibril-forming motifs VQIVYK [37]. Considering that parietin has groups with a negative charge density, we proposed that its activity against aggregation process of tau is due to molecular interactions with fibril-forming motifs. In the complex between orange-G and VQIVYK, the fragment of tau has a β-sheet form with the dye binding between two sheets. Considering this information, we constructed the possible tridimensional structure of parietin in interaction with the tau VQIVYK motif using docking. Since we do not have information about the preferred protonation state of parietin forming the complex, we tested the protonation states P1 and P2 described in Materials and Methods section. We docked parietin structures inside a cavity formed between two steric zippers (model A) and on the surface of one steric zipper (model B); models A and B (Figure 3).

Docking results for parietin in protonation states P1 and P2 inside the model A, shown in Figure 3A. The obtained models suggest that parietin could serve as inhibitor of aggregation by binding between steric zippers preventing higher-order β-sheet interactions as orange-G [37]. The phenolic groups and the oxygen of methoxy substituent form hydrogen bond (HB) interactions with different VQIVYK lysine side chains. At once, the methyl group of the methoxy substituent has hydrophobic interactions with the VYK valine. It is noteworthy that the results are comparable for both protonation states of parietin.

The docking results for parietin in protonation states P1 and P2 inside the model B are shown in Figure 3B. The models obtained show that both protonation states have similar orientations forming HB interactions with lysine and glutamine side chains. Phenolic groups and carbonyl groups at position 9 of the anthracene-9,10-dione scaffold have HB interactions with glutamine side chains, while the oxygen of methoxy substituent and carbonyl at position 10 of the anthracene-9,10-dione scaffold have HB interactions with lysine side chains. At the same time, methyl groups orient near VYK valine forming hydrophobic interactions.

Regarding the HPLC fingerprint of the methanolic extract, 22 compounds were identified for the first time in the methanolic extract of *R. terebrata* with the help of their characteristic UV-Vis spectra and high-resolution mass spectrometry [38,39]. All compounds were detected in negative mode using UHPLC-Q/Orbitrap/ESI/MS/MS (Table 1). Peak 22 was identified as parietin (molecular anion at *m*/*z* 283.0601). Peak 21 was identified as usnic acid, which showed a [M − H]^−^ peak at *m*/*z* 343.0803. Major diagnostic daughter MS ions of usnic acid were [M − H − CH_3_]^−^, [M − H − C_4_H_3_O_2_]^−^ and [M − H − C_5_H_3_O_3_]^−^ (328.0583, 259.0612 and 231.0663 amu, respectively). Peak 20 was identified as lobaric acid (molecular anion at *m*/*z* 455.1712). The fragmentation of peak 20 also produced ions at 411.1808 [M − H − CO_2_]^−^, 367.1909 [M − H − 2CO_2_]^−^, 352.1675 [M − H − 2CO_2_ − CH_3_]^−^, and 296.1049 [M − H − 2CO_2_ − C_5_H_11_]^−^ confirming this depsidone. Peak 19 and 17 had the same [M − H]^−^ ion at *m*/*z* 375.1070 with different retention time based on UHPLC at 22.04 and 23.65 min, which were tentatively identified as placodiolic acid or pseudoplacodiolic acid, respectively. Peak 18 with a [M − H]^−^ ion at *m*/*z* 527.2290 was identified as arthoniaic acid, and peak 16 as gyrophoric acid, which was identified by spiking experiments with an authentic standard. Peak 15 with a [M − H]^−^ ion at *m*/*z* 497.1065 was identified as 3-hydroxyumbilicaric acid. Main daughter ion of peak 15 was at *m*/*z* 317.0652 [M − H − C_9_H_8_O_4_]^−^. Peak 8 could be tentatively identified as 4-*O*-dimethylbaemycesic acid (*m*/*z* 359.0756) which produced a MS^2^ ion at *m*/*z* 302.0417. Ten tetrahydroxy fatty acids (peak 1–3, 5, 7, 9–11 and 13–14) and three pentahydroxy fatty acids (peak 4, 6 and 12) were tentatively identified as the polihydroxy fatty acids reported by Huneck [30].

On the other hand, in recent years, some chemical studies belonging to Ramalina genus have been published and most of the works have been focused on secondary metabolites [40]. *Ramalina terebrata* Hook and Taylor from the Antarctic is the producer of usnic acid, ramalin, stereocalpin A and usimines A–C [40,41,42,43]. Besides, it has been reported from the Ramalina genus isousnic acid, usninic acid, the following depsides sekikaic acid and its 5-OH, 5-Cl derivatives, 4′-*O*-methylsekikaic acid, 4′-*O*-demethylsekikaic acid, 4′-*O*-methylnorsekikaic, 2′-*O*-methylsekikaic, homosekikaic acid, 4′-*O*-methylnorhomosekikaic acid, 4′-*O*-demethylhomosekikaic acid, atranorin, chloroatranorin, divaricatic acid, ramalinolic acid, obtusatic acid, chlorotumidulin, evernic acid, diffractaic acid, 4′-*O*-demethylbarbatic acid, ramalinaic acid, cryptochlorophaeic acid and its 4,4′-dimethyl derivative, gyrophoric acid, trivaric acid, perlatolic acid, 4′-*O*-methylpaludosic acid, boninic acid, stenosporic acid, olivetoric acid, paludosic acid, lecanoric acid, and bourgeanic acid. Also, the following depsidones salazinic acid, norstictic acid, hypoprotocetraric acid, conhypoprotocetraric acid, scopuloric acid, protocetraric acid, connorstictic acid, cryptostictic acid, peristictic acid, variolaric acid, gangaleoidin, physodic acid, and coquimboic acid have been isolated from Ramalina genus. Finally, the fatty acids reported from Ramalina were oleic, palmitic, stearic, linolenic, linoleic, and myristic acids, and the γ-lactone acids protolichesterinic, d-protolichesterinic, and nephrosterinic [40].

Some lichen extracts from the genus Ramalina have displayed a wide range of biological activities such as antimicrobial, antioxidant, antiviral, antitumoral, cytotoxicity, antiinflammatory, and antihelmintic [30,40,41,42,43,44,45,46]. An acetone extract of *R. farinacea* demostrated activity against *Candida albicans* and *Candida glabrata* at concentrations ranging between 3.3 to 6.6 µg/25 µL. Furthemore, a methanolic extract of *R. pollinaria* showed antibacterial activity and presented MIC values between 5.62–62.5 µg/µL, while the MIC values for *R. polymorpha* was 62.5 µg/µL. Cansaran [44] studied five Ramalina species, and among them the methanolic extract of *R. fastigiata* showed the best inhibition against *Bacillus subtilis*, *Enterococcus faecalis*, *Escherichia coli* and *Proteus mirabilis*. Methanolic extract from *R. hossei* showed better activity against Gram(+) than against Gram(−) bacteria [45]. The hexanic extract from *R. roesleri* showed a high activity against *S. aureus* and *S. mutans*. In other study, the antibacterial activity of methanolic extract of Antarctic lichen *R. terebrata* displayed considerable antimicrobial activity against *Bacillus subtilis* (MIC 33.8 ± 0.15 µg/µL) and *S. aureus* (MIC 85.7 ± 6.7 µg/mL) but no activity against *C. Albicans*, *P. aeruginosa*, and *E. coli*, while Paudel et al. [46] reported activity against *S. aureus*. Regarding to antioxidant activity, the methanolic extracts of *R. pollinaria* and *R. polymorpha* did no show antioxidant properties based on the DPPH method. However, a low inhibition was showed on the oxidation of linoleic acid/β-carotene method. The methanolic extract of *R. hossei* and *R. conduplicans* displayed antioxidant potential by the DPPH method and by the reduction of Fe^+3^ assay. On the other hand, an acetonic extract of *R. peruviana* presented antioxidant activity on the DPPH method (86%) and β-carotene assay (57.3%). A ethanol-water extract (1:1) of *R. capitata* displayed gastroprotective activity (66%) at a dose of 200 mg/kg on the gastric damage model induced by indomethacin, while a methanolic extract of *R. cuspidata* shows interesting cytotoxic activity particulary in the cell lineages K-562, U251, DU145 and MCF7 [40].

Regarding the lichen-derived substances, usimine C has showed anti-proliferative activity on human dermal fibroblasts [41]. Usnic acid, usimine A–C and ramalin isolated from Antarctic lichen *R. terebrata*, displayed activity against *B. subtilis* but no activity against *S. aureus*, where the values of MIC ranged from 1–26 μg/mL [40]. Ramalin showed to be a more powerful antioxidant than butylhydroxytoluene (BHT), vitamin E, Trolox and ascorbic acid as well as a very good antiinflammatory agent [43]. Sekikaic acid and homosekikaic acid showed IC_50_ values of 0.082 mg/mL and 0.276 mg/mL at the linoleic acid peroxidation assay, demonstrating that these compounds are promising antioxidants. The antioxidant activity of atranorin, protolichesterinic acid, usnic acid, 2-hydroxy-4-methoxy-6-propylbenzoic acid, homosekikaic acid, sekikaic acid, 2,4-dihydroxy-6-propylbenzoic acid and 2,4-dihydroxy-3,6-dimethylbenzoate isolated from the hexane extract from *R. roesleri* were assessed by the DPPH method. Among the compounds, the best antioxidant activity was exhibited by sekikaic acid, followed by homosekikaic acid. Usnic acid has demostrated to reduce the production of Junin virus in infected Vero cells in a dependent dose manner (EC_50_ 9.9 μM), these results indicate that usnic acid present antiviral activity. Also, usnic acid is not genotoxic and cytotoxic towards human lymphocyte A549, promyelocytic leukemia HL-60, and ovarian carcinoma A2780. d-protolichesterinic acid and nephrosterinic acid showed activity against Ehrlich carcinoma. d-protolichesterinic acid and lobaric acid are considered 5- and 12-lipoxygenase inhibitors [40].

## 3. Materials and Methods

### 3.1. Collection and Identification of Lichen Species

*Ramalina terebrata* was collected in “Peninsula Fildes” Antarctic region, Chile during March, 2014. Lichens were carefully removed from the rocks by abrasion. A voucher specimens were deposited at the Extreme Natural Product laboratory, Universidad de Chile whose reference numbers is RT-010414. *R. terebrata* (50 g) were dried, powdered and extracted with methanol (3 × 0.5 L) to afford, after evaporation of the solvent under vacuum, 450 mg of a dark gum.

### 3.2. Extraction

The methanolic extract was subjected to Sephadex LH-20 and eluted with MeOH. Fractions (10 mL) were monitored by TLC and combined to give two main fractions A–B. Further purification was done for the fractions A and B. Fraction A (200 mg) was chromatographed on silica gel (50 g, 63–200 µm) using n-hexane/EtOAc mixtures (0% to 100%) giving 30 mg of parietin **1** [30] and 54 mg of usnic acid **2** [30]. Fraction B (110 mg) was chromatographed on silica gel (30 g, 63–200 µm) using DCM/MeOH mixtures (0% to 100%) giving 10 mg of parietin **1**, 15 mg of atraric acid **3** [30] and 10 mg of inositol **4** [30].

### 3.3. Tau Protein Production

Tau fragment 4R (htau_244-372_) was amplified by using the plasmid for htau40 as a template. The PCR sequence was subcloned into pET-28a vector (Novagen, Madinson, WI, USA) to produce a His-tagged protein. The recombinant fragment 4RMBD was expressed in *Escherichia coli* strain BL21 (DE3) as described [29]. LB medium containing kanamycin was inoculated with a stationary overnight culture. Bacterial culture was grown at 37 °C to OD_600_ of 0.5–0.6 and protein expression was induced by addition of 1 mM IPTG for 4 h; and cells were pelleted and sonicated. Recombinant tau was purified via a succession of Ni-Sepharose chromatography (equilibrated in 20 mM NaH_2_PO_4_, 500 mM NaCl, and 20 mM imidazole, pH 7.4, elution with buffer 200 mM imidazole) and side exclusion chromatography coupled to HPLC in a Proteema 100 column (PSS, Mainz, Germany) with buffer 50 mM NaH_2_PO_4_, 300 mM NaCl, pH 6.5. The purity of the protein was verified on a Coomassie Brilliant Blue-stained SDS-polyacrylamide gel. The protein was concentrated and stored at −80 °C until use. The concentration of purified 4RMBD was determined using the extinction coefficient at 280 nm (1520 M^−1^·cm^−1^).

### 3.4. Thioflavin T Assay

The ThT fluorescence assay adopted here was modified from the reported by Pickhardt et al. [36]. Briefly, to examine the inhibition of tau aggregation, the total volume of the reaction mixture was 100 μL, which included 20 μM 4RMBD, 5 μM heparin in 100 mM sodium acetate, pH 7.0 with parietin at different concentrations. After 20 h of incubation at 37 °C, addition of 100 μL of a 25 μM solution of ThT was made and incubation continued for 1 h at room temperature prior to fluorescence reading. Then, the fluorescence was measured in a Biotek H1 multi-mode reader (Biotek Instruments, Winooski, VT, USA) with an excitation wavelength at 440 nm and emission wavelength of 485 nm in a 96-well plate. Each experiment was replicated at least three times and background fluorescence was subtracted.

### 3.5. UHPLC-Q/Orbitrap/MS/MS

#### 3.5.1. Instrument

A Thermo Scientific Dionex Ultimate 3000 UHPLC system equipped with a quaternary Series RS pump and a Thermo Scientific Dionex Ultimate 3000 Series TCC-3000RS column compartments with a Thermo Fisher Scientific Ultimate 3000 Series WPS-3000RS autosampler and a rapid separations PDA detector controlled by Chromeleon 7.2 Software (Thermo Fisher Scientific, Bremen, Germany) hyphenated with a Thermo high resolution Q Exactive focus mass spectrometer were used for analysis. The chromatographic system was coupled to the MS with a Heated Electrospray Ionization Source II (HESI II). Nitrogen (purity > 99.999%) obtained from a Genius NM32LA nitrogen generator (Peak Scientific, Billerica, MA, USA) was employed as both the collision and damping gas. Mass calibration for Orbitrap was performed once a week, in both negative and positive modes, to ensure a working mass accuracy lowers than or equal to 5 ppm. Cafeine and *N*-butylamine (Sigma Aldrich, Saint Louis, MO, USA) were the calibration standards for positive ions and buspirone hydrochloride, sodium dodecyl sulfate, and taurocholic acid sodium salt were used to calibrate the mass spectrometer. These compounds were dissolved in a mixture of acetic acid, acetonitrile, water and methanol (Merck Darmstadt, Hesse, Germany) and were infused using a Chemyx Fusion 100 syringe pump. XCalibur 2.3 software and Trace Finder 3.2 (Thermo Fisher Scientific, San Jose, CA, USA) were used for UHPLC control and data processing, respectively. Q Exactive 2.0 SP 2 from Thermo Fisher Scientific was used to control the mass spectrometer.

#### 3.5.2. LC Parameters

Liquid chromatography was performed using an UHPLC C18 column (Acclaim, 150 mm × 4.6 mm ID, 5 m) operated at 25 °C. The detection wavelengths were 254, 280, 320 and 440 nm, and PDA was recorded from 200 to 800 nm for peak characterization. Mobile phases were 1% formic aqueous solution (A) and acetonitrile (B). The gradient program (time (min), %B) was: (0.00, 5); (5.00, 5); (10.00, 30); (15.00, 30); (20.00, 70); (25.00, 70); (35.00, 5) and 12 min for column equilibration before each injection. The flow rate was 1.00 mL·min^−1^, and the injection volume was 10 µL. Standards and lichen extracts dissolved in methanol were kept at 10 °C during storage in the autosampler.

#### 3.5.3. MS Parameters

The HESI parameters were optimized as follows: sheath gas flow rate 75 units; aux. gas unit flow rate 20; capillary temperature 400 °C; aux gas heater temperature 500 °C; spray voltage 2500 V (for ESI^−^); and S lens RF level 30. Full scan data in both positive and negative was acquired at a resolving power of 70,000 FWHM (full width half maximum) at *m*/*z* 200. For the compounds of interest, a scan range of *m*/*z* 100–1000 was chosen; the automatic gain control (AGC) was set at 3 × 10^6^ and the injection time was set to 200 ms. Scan-rate was set at 2 scans·s^−1^. External calibration was performed using a calibration solution in positive and negative modes before each sample series. In addition to the full scan acquisition method, for confirmations purposes, a targeted MS/MS analysis was performed using the mass inclusion list and expected retention times of the target analytes, with a 30 s time window, with the Orbitrap spectrometer operating both in positive and negative mode at 17,500 FWHM (*m*/*z* 200). The AGC target was set to 2 × 10^5^, with the maximum injection time of 20 ms. The precursor ions are filtered by the quadrupole which operates at an isolation window of *m*/*z* 2. The fore vacuum, high vacuum and ultrahigh vacuum were maintained at approximately 2 mbar, from 105 and below 1010 mbar, respectively. Collision energy (HCD cell) was operated at 30 kV. Detection was based on calculated exact mass and on retention time of target compounds, as shown in Table 1. The mass tolerance window was set to 5 ppm for the two analysis modes.

### 3.6. Molecular Modeling

The current structural information about tau protein is very limited; however, our results suggest that parietin could interact with tau fibril-forming motifs VQIINK (PHF6*), particularly with lysine residues. The coordinates of the hexapeptide VQIVYK were extracted from the X-ray crystal structure of tau VQIVYK segment complexed with orange-G (Protein Data Bank code 3OVL) [47,48]. The fiber structure was prepared using the VQIVYK coordinates. Zinc atoms were removed since zinc solution was not used in our study. We prepared two fiber structure models A and B: A contains 24 units of hexapeptide and B contains 12 units of the hexapeptide. In model A, each member of a pair of VQIVYK β-sheets is shifted relative to the other, without the dry interface in the typical steric zipper structure, forming a cylindrical cavity [47,48]; docking was executed inside the cavity. The model B just contains the half of peptides of model A, and B does not contain a cavity; therefore, the docking was executed at surface. Using models A and B, we evaluated the interactions between the parietin and the VQIVYK fiber structure using these models considering the presence and absence of a cavity.

For docking, we considered two protonation states of parietin: P1 with neutral phenolic groups, and P2 with deprotonated phenolic groups. These two extreme cases allow studying the effect of the protonation state of molecular interactions with hexapeptide VQIVYK. Both models of parietin (P1 and P2) were sketched using Maestro’s molecular editor.

Docking was performed using Glide method [47,48]. A grid box of 15 Å × 10 Å × 10 Å covered the whole cavity in model A, and whole surface in model B. Docking parameters were used as previously reported, a Glide extra-precision (XP) modes were explored during search. Docking hierarchy begins with systematic conformational expansion of ligand followed by placement on receptor site. Then, minimization of the ligand in the receptor field was carried out using the OPLS-AA [49] force field with a distance-dependent dielectric of 2.0. Afterwards, the lowest energy poses were subjected to a Monte Carlo procedure that samples the nearby torsional minima. The best pose for a given ligand was determined by the Emodel score, while different compounds were ranked using GlideScore [50]. Docking poses were analyzed by examining their relative total energy score. The most energetically favorable conformations were selected as best poses.

### 3.7. Statistical Analysis

Results of statistical analysis were expressed as the mean ± SEM. In all experiments, statistical differences between treatments and their respective control were determined by Paired *t*-test. Significance level was set at *p* < 0.05. All statistical analyses were developed using GraphPad Prism 6 software (H. Motulsky, San Diego, CA, USA).

## 4. Conclusions

Finally, our results demonstrate that parietin has a moderate activity against the aggregation process of tau protein, while the methanolic extract of *Ramalina terebrata* had also activity that could be attributed to sinergistic effects of the compounds detected in the extract. On the other hand, docking experiments suggest that parietin is bound to fiber-forming segment VQIVYK of tau mediated mainly by HBs interactions with the lysine residues. Based on UHPLC-Q/Orbitrap/ESI/MS/MS, 22 compounds were identified in the methanolic extract of the Antarctic lichen *Ramalina terebrata*. Finally, in-depth analysis of the chemical composition of *R. terebrata* could guide further research into its medicinal properties and potential uses.

## Figures and Tables

**Table 1:** Identification of metabolites in Antarctic lichen *R. terebrata* by UHPLC-Q/Orbitrap/ESI/MS/MS. * Identified by spiking experiments with an authentic compound; retention time (min); theoretical and measured mass (*m*/*z*); accuracy (ppm).

Peak	Tentative Identification	[M–H]^−^	Retention Time	Theoretical Mass	Measured Mass	Accuracy	MS^n^ Ions
1	9,10,12,13-tetrahydroxyheptadecanoic acid	C_17_H_33_O_6_	14.53	333.2283	333.2267	4.8	–
2	9,10,12,13-tetrahydroxyoctadecanoic acid	C_18_H_35_O_6_	15.54	347.2439	347.2423	4.6	–
3	9,10,12,13-tetrahydroxynonadecanoic acid	C_19_H_37_O_6_	17.45	361.2596	361.2577	5.2	343.2472
4	9,10,11,12,13-pentahydroxydocosanoic acid	C_22_H_43_O_7_	18.54	419.3014	419.2995	4.5	–
5	9,10,12,13-tetrahydroxyeicosanoic acid	C_20_H_39_O_6_	18.61	375.2752	375.2736	4.2	357.2628; 187.0962
6	9,10,11,12,13-pentahydroxytricosanoic acid	C_23_H_45_O_7_	19.21	433.3165	433.3150	3.5	–
7	9,10,12,13-tetrahydroxyheneicosanoic acid	C_21_H_41_O_6_	19.29	389.2909	389.2890	4.8	371.2782
8	4-O-dimethylbaemycesic acid	C_18_H_15_O_8_	19.58	359.0767	359.0756	3.0	–
9	9,10,12,13-tetrahydroxyeicosanoic acid	C_20_H_39_O_6_	19.64	375.2747	375.2735	3.2	–
10	9,10,12,13-tetrahydroxydocosanoic acid	C_22_H_43_O_6_	19.80	403.3065	403.3047	4.4	385.2940; 215.1274
11	9,10,12,13-tetrahydroxyheneicosanoic acid	C_21_H_41_O_6_	19.95	389.2909	389.2892	4.3	371.2782
12	9,10,11,12,13-pentahydroxytetracosanoic acid	C_24_H_47_O_7_	20.20	447.3327	447.3306	4.7	–
13	9,10,12,13-tetrahydroxydocosanoic acid	C_22_H_43_O_6_	20.37	403.3065	403.3043	5.4	385.2938; 187.0961
14	9,10,12,13-tetrahidroxytricosanoic acid	C_23_H_45_O_6_	20.79	417.3222	417.3198	5.7	399.3094
15	3-hydroxyumbilicaric acid	C_25_H_21_O_11_	21.25	497.1089	497.1065	4.8	317.0652; 167.0336
16	Gyrophoric acid *	C_24_H_19_O_10_	21.27	467.0978	467.0962	3.4	317.0647; 167.0336; 149.0230; 123.0438
17	Placodiolic acid or Pseudoplacodiolic acid	C_19_H_19_O_8_	22.04	375.1079	375.1070	2.4	343.0807; 259.0598; 231.0648
18	Arthoniaic acid	C_29_H_36_O_9_	22.78	527.2281	527.2290	−1.7	–
19	Pseudoplacodiolic acid or Placodiolic acid	C_19_H_19_O_8_	23.65	375.1079	375.1068	2.9	343.0805; 259.0597; 231.0647
20	Lobaric acid *	C_25_H_27_O_8_	24.82	455.1711	455.1712	−0.2	411.1808; 367.1909; 352.1675; 296.1049
21	Usnic acid *	C_18_H_15_O_7_	26.17	343.0818	343.0803	4.3	328.0583; 259.0612; 231.0663
22	Parietin	C_16_H_11_O_5_	27.21	283.0612	283.0601	3.9	–

## Supplementary Materials

Supplementary materials can be found at www.mdpi.com/1422-0067/17/8/1303/s1.
