# Supplementary material for: Secondary Metabolites in Ramalina terebrata Detected by UHPLC/ESI/MS/MS and Identification of Parietin as Tau Protein Inhibitor
Source: Int J Mol Sci. 2016 Aug 18;17(8):1303. doi: 10.3390/ijms17081303 (PMC5000700; doi:10.3390/ijms17081303)
Supplement: Supplementary file 1 [file ijms-17-01303-s001.pdf]

Supplementary Materials: Secondary Metabolites in *Ramalina Terebrata* Detected by UHPLC/ESI/MS/MS and Identification of Parietin as Tau Protein Inhibitor

Alberto Cornejo, Francisco Salgado, Julio Caballero, Reynaldo Vargas, Mario Simirgiotis and Carlos Areche

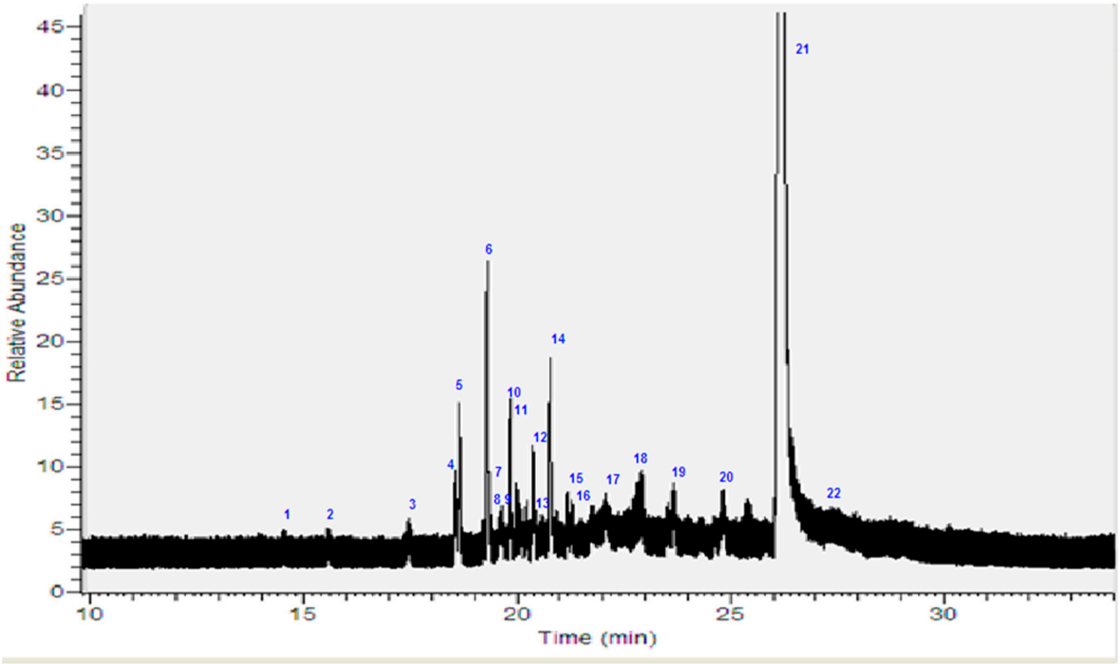

Figure S1. UHPLC-PDA chromatograms of the methanolic extract of *R. terebrata*. Peaks numbers refer to those indicated in Table 1.

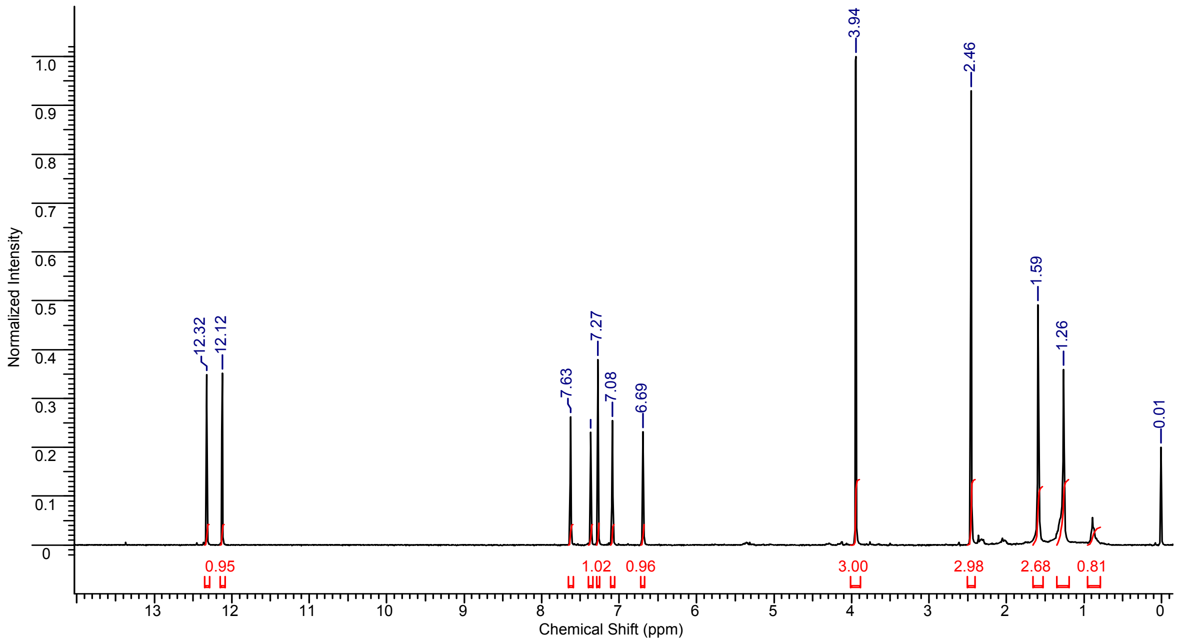

Figure S2. ^{1}H-NMR spectrum of parietin 1 in $CDCl_3$ (400 MHz).

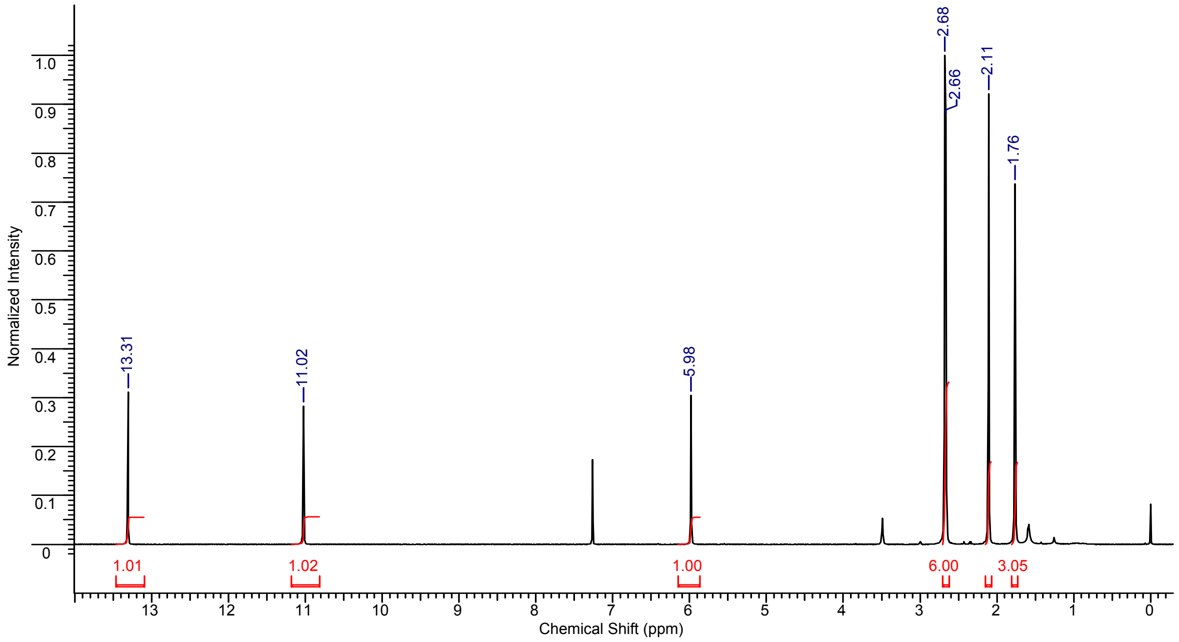

Figure S3. ^{1}H-NMR spectrum of usnic acid 2 in $CDCl_3$ (400 MHz).

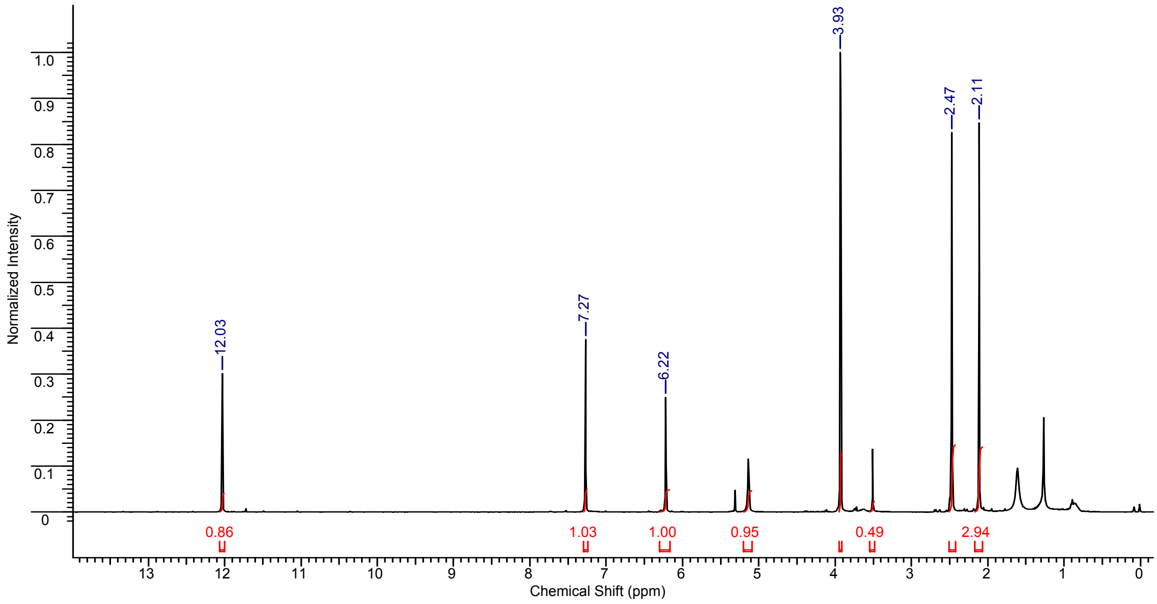

Figure S4. ^{1}H-NMR spectrum of 3 in $CDCl_3$ (400 MHz).

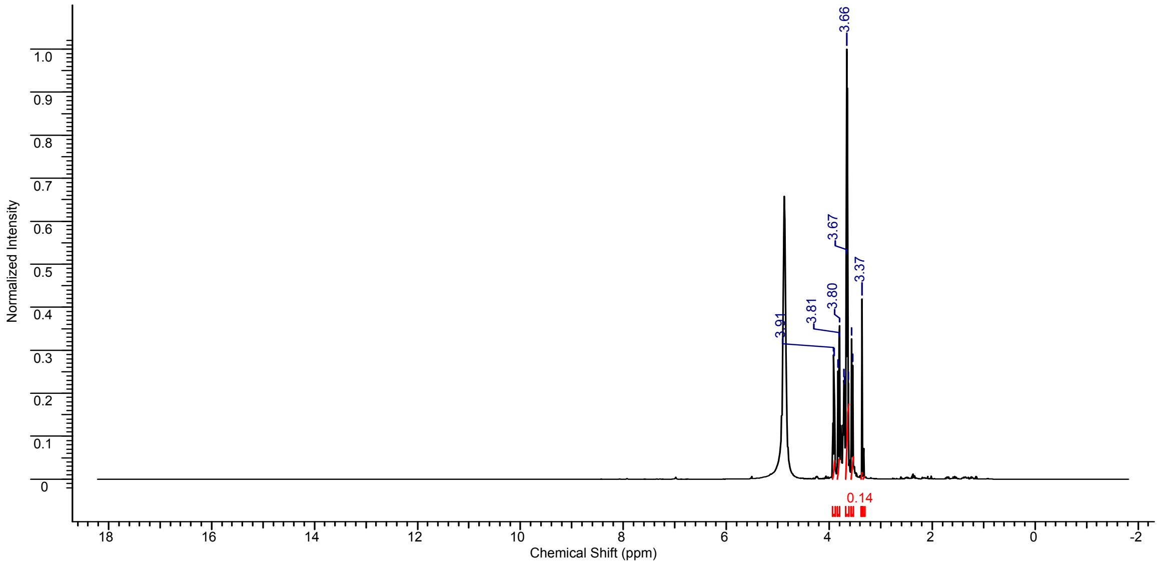

Figure S5. ^{1}H-NMR spectrum of 4 in $MeOD_4$ (400 MHz).

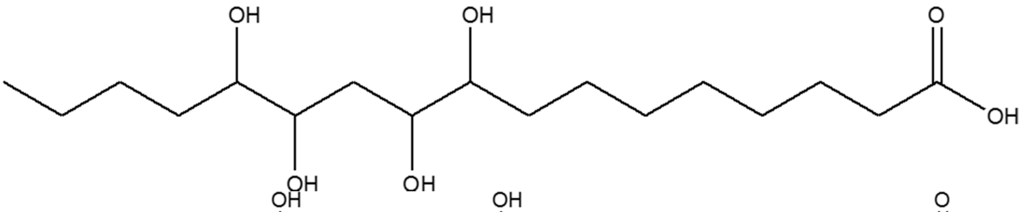

Peak 1

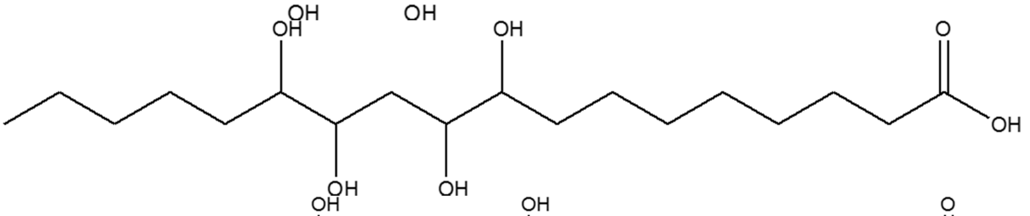

Peak 2

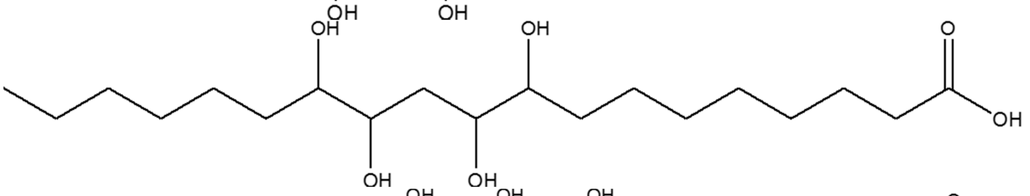

Peak 3

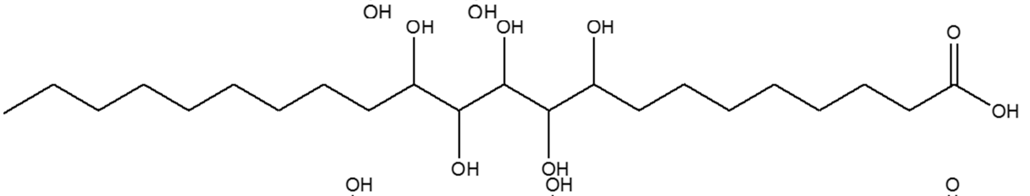

Peak 4

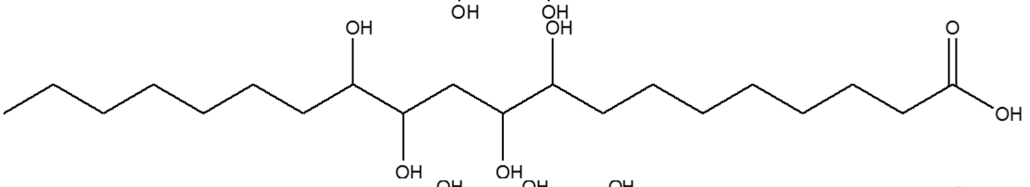

Peak 5

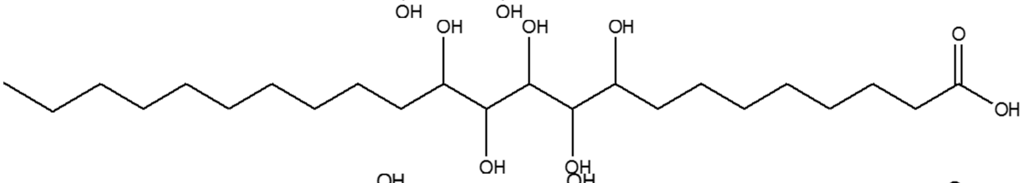

Peak 6

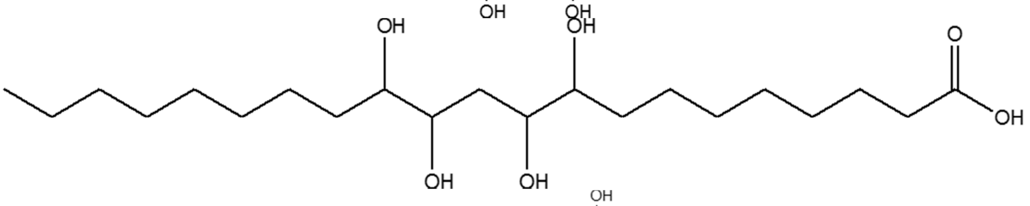

Peak 7

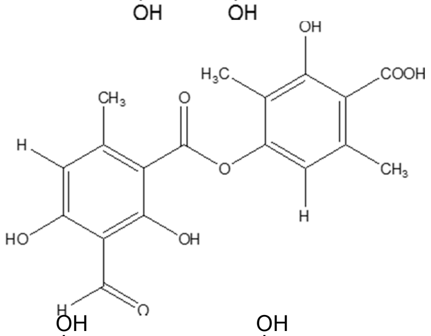

Peak 8

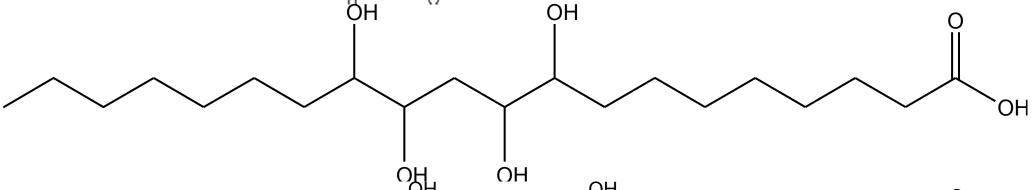

Peak 9

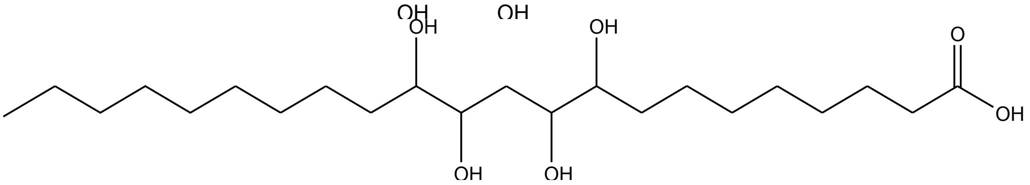

Peak 10

Figure S6. *Cont.*

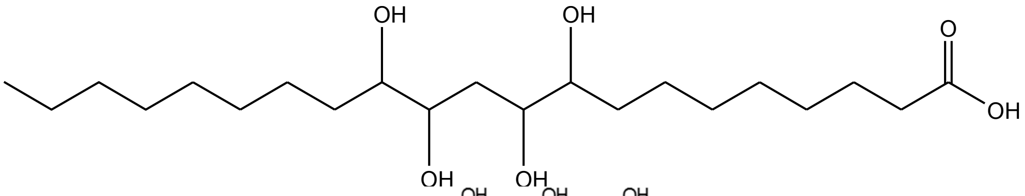

Peak 11

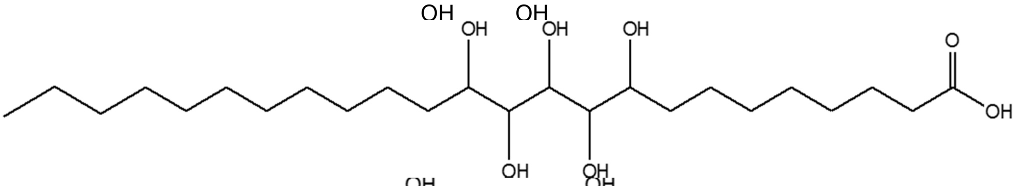

Peak 12

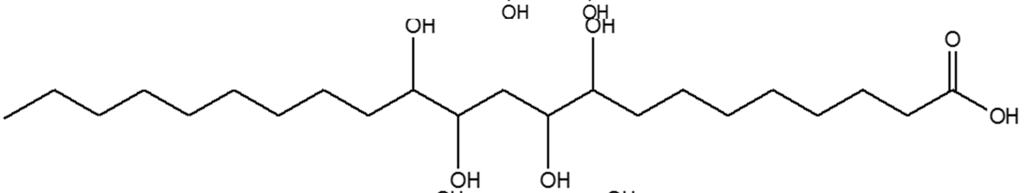

Peak 13

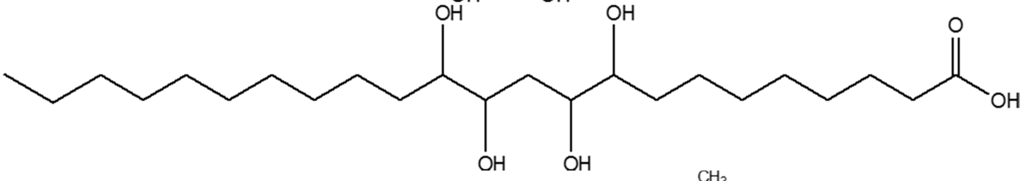

Peak 14

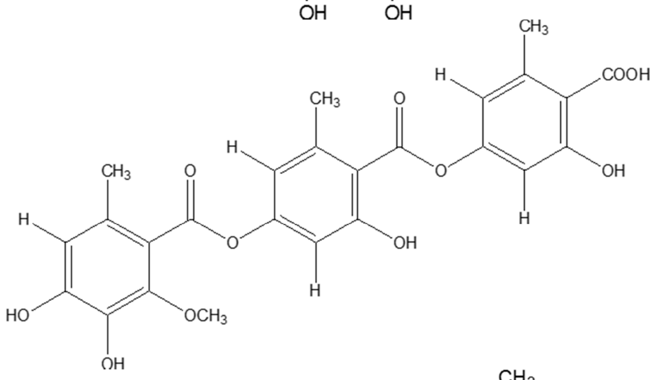

Peak 15

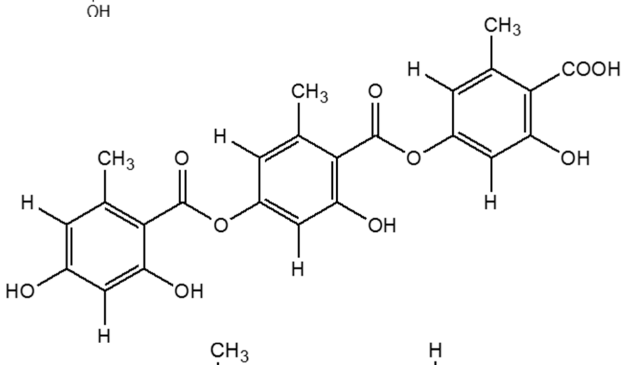

Peak 16

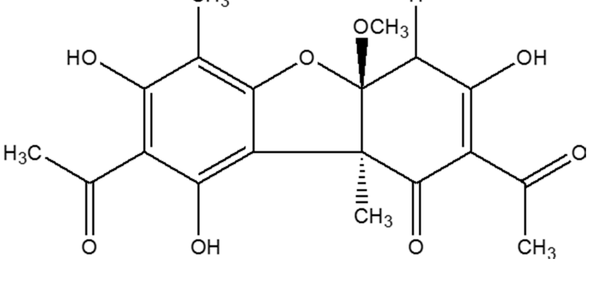

Peak 17

Figure S6. *Cont.*

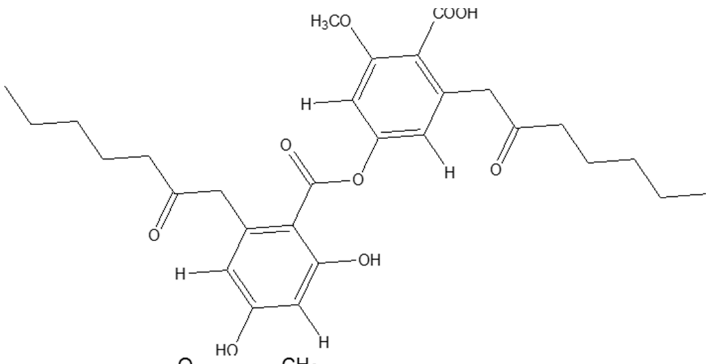

Peak 18

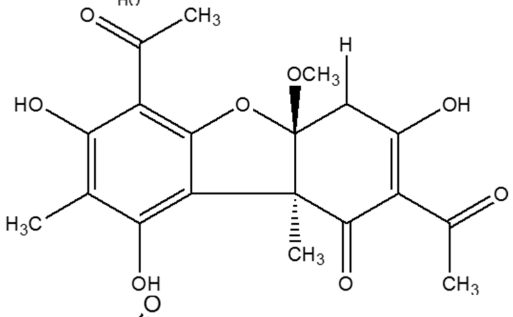

Peak 19

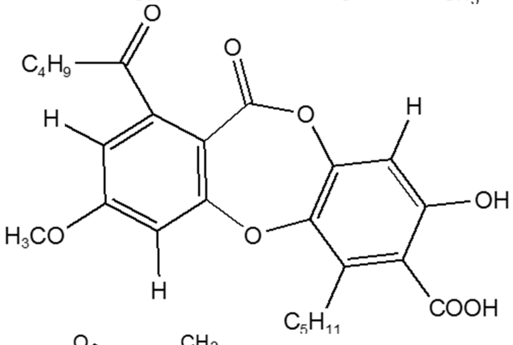

Peak 20

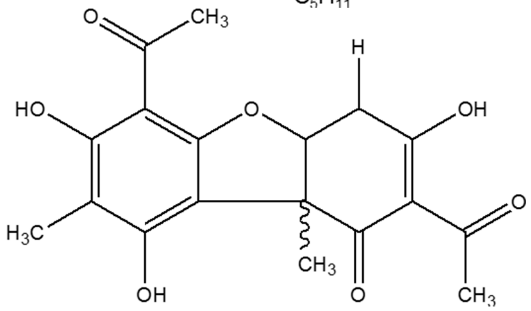

Peak 21

Figure S6. Chemical structures of the compounds identified by UHPLC/ESI/MS/MS.
